# Exposed Areas Above Sea Level on Earth >3.5 Gyr Ago: Implications for Prebiotic and Primitive Biotic Chemistry

**DOI:** 10.3390/life8040055

**Published:** 2018-11-04

**Authors:** Jeffrey L. Bada, Jun Korenaga

**Affiliations:** 1 Scripps Institution of Oceanography, University of California, San Diego, CA 92093, USA; 2 Department of Geology and Geophysics, Yale University, New Haven, CT 06520, USA; jun.korenaga@yale.edu

**Keywords:** continental crust, exposed land, prebiotic chemistry, volcanic islands, volcanic lightning

## Abstract

How life began on Earth is still largely shrouded in mystery. One of the central ideas for various origins of life scenarios is Darwin’s “warm little pond”. In these small bodies of water, simple prebiotic compounds such as amino acids, nucleobases, and so on, were produced from reagents such as hydrogen cyanide and aldehydes/ketones. These simple prebiotic compounds underwent further reactions, producing more complex molecules. The process of chemical evolution would have produced increasingly complex molecules, eventually yielding a molecule with the properties of information storage and replication prone to random mutations, the hallmark of both the origin of life and evolution. However, there is one problematic issue with this scenario: On the Earth >3.5 Gyr ago there would have likely been no exposed continental crust above sea level. The only land areas that protruded out of the oceans would have been associated with hotspot volcanic islands, such as the Hawaiian island chain today. On these long-lived islands, in association with reduced gas-rich eruptions accompanied by intense volcanic lightning, prebiotic reagents would have been produced that accumulated in warm or cool little ponds and lakes on the volcano flanks. During seasonal wet–dry cycles, molecules with increasing complexity could have been produced. These islands would have thus been the most likely places for chemical evolution and the processes associated with the origin of life. The islands would eventually be eroded away and their chemical evolution products would have been released into the oceans where Darwinian evolution ultimately produced the biochemistry associated with all life on Earth today.

## 1. Introduction

Although Charles Darwin is famous for his masterpiece “*The Origins of the Species*” (1859), he is also well known for his concept of a “warm little pond” (WLP) and its possible role in abiogenesis. Darwin was at first reluctant to address the question of the origin of life [1], noting in the 3rd edition of “*Origins*” (1861) “it is mere rubbish thinking, at present of origin of life”. He never directly addressed the idea of a “warm pond” in his formal published writing. Yet, he obviously thought about the origin of life issue a great deal. In his classic 1871 letter to his friend Joseph Dalton Hooker he noted “But if (and oh what a big if) we could conceive in some warm little pond” [1].

Nearly a century and a half later, WLPs (also Cool Little Ponds) remains a central concept with respect to our understanding of the possible environments on the early Earth [and elsewhere] that might have been associated with prebiotic chemistry and the transition to primitive biotic chemistry [for example see [2,3,4,5]. However, as Deamer recently noted “so little is known with certainty about the late Hadean Era” [6]. In this context, a significant issue is what were the amounts and types of exposed areas above sea level, and what was their stability, on Earth >3.5 Ga. If exposed areas above sea level were scarce, were WLPs indeed plausible on the early Earth? This is a critical issue because it was in this period that life arose based on microfossil and geochemical fossil evidence [7,8].

Without exposed continental areas, the early oceans would have been the main reservoir of the prebiotic compounds thought to be involved in the transition from prebiotic to biotic chemistry. This presents a conundrum, however. Oceanic concentrations of the relevant prebiotic molecules from whatever sources would have been likely low. For example, Miller estimated that the early ocean maximum hydrogen cyanide (HCN) concentration, an important reagent in a variety of prebiotic syntheses, was ~4 µM based on the stability, production, and destruction of HCN during circulation through hydrothermal vents [9]. However, even this steady-state concentration of HCN could have in turn yielded a steady state total amino acid concentration of ~0.3 mM, hydrothermal vent circulation again being the limiting factor. Yet is unknown whether geologically rapid hydrothermal circulation [the entire oceans passed through the vents in 10 Myr today] existed on the early Earth before the origin of modern plate tectonics. If the hydrothermal destructive process was not as effective as it is today, the HCN in the ocean could have been higher, perhaps making the formation of prebiotic compounds less problematic.

The infall of carbonaceous meteorites and their associated organic compounds could have also contributed prebiotic compounds to the early oceans. But the survival of extraterrestrial organic molecules, mainly in the form of interplanetary dust particles (IDPs), during atmospheric passage and heating is a major concern [10,11]. As was the case with prebiotic compounds made directly on Earth, oceanic extraterrestrial compounds accumulation could have been limited by hydrothermal circulation if it existed.

Early “hotspot” volcanic islands may have been more important as areas that extruded above sea level [12]. Unlike mid-ocean-ridge magmatism or arc magmatism, hotspot magmatism does not require the operation of plate tectonics, and it is generally considered to represent the bulk of endogenous magmatism on other terrestrial planets in the solar system. Likely hotspot volcanic islands on the early Earth, with the ubiquitous volcanic lightning associated with their eruptions, and warm ponds or lakes on the volcano flanks, appear to be plausible areas for the effective prebiotic chemistry that set the stage of the emergence of the first primitive biotic chemistry.

Another important consideration is the chemistry of any WLPs or lakes on exposed land areas on early Earth. These may have had corrosive conditions (for example slightly acidic pH, reduced metal ion contents, etc.) that could have limited the survival of prebiotic compounds. As noted by Saito et al. [13], the early oceans would have had high concentrations of Fe^+2^ and “the relative availability of trace metals would have been similar to that of a sulfidic system, Fe > Mn, Ni, Co >> Cd, Zn, Cu…”. If the chemistry of WLPs also reflected this overall composition, how these dissolved components affect prebiotic syntheses and prebiotic compound stability needs to be addressed.

## 2. Early Exposed Areas Above Sea Level

To discuss exposed land masses on the early Earth, we first need to understand the following two notions: (1) the area of exposed continent crust is different from the mass of continental crust, and (2) plate tectonics, which affect almost all aspects of modern geological processes including the generation of continental crust, may have been absent on early Earth.

There exist a number of different models for the growth of continental crust [14,15,16,17,18,19], but they are all about the history of the mass (or volume) of continental crust, with little information on what fraction of continental area was above sea level in the past. For example, even if the continental crust at 4 Gyr ago was as massive as the present-day crust, it could have been all under water if the volume of oceans were sufficiently greater than present. Indeed, early continental crust is likely to have been submerged, as is discussed later. Pearce et al. [2] used a linear growth model to estimate exposed continental crust between 4.5 and 3.7 Gyr ago, which yields at 3.7 Gyr ago a value of 12.5% modern or ~20 × 10^6^ km^2^. As discussed below, this is likely a large overestimate.

**Figure 1:**
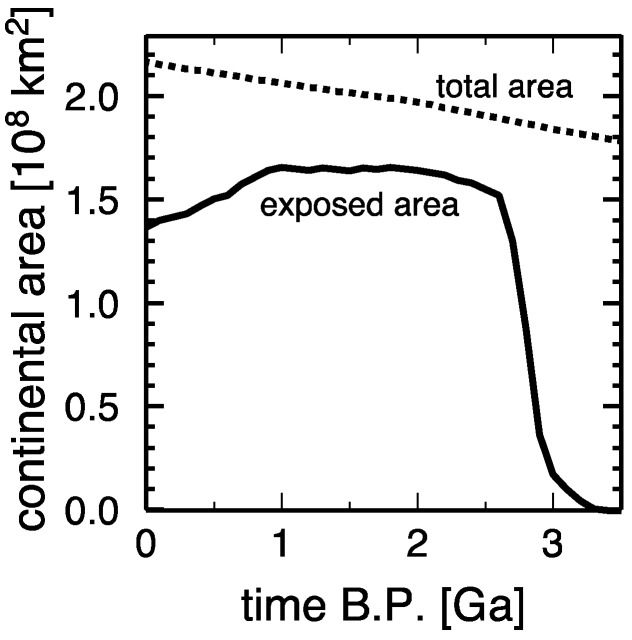
Likely evolution of continental area on the Earth based on continental freeboard modeling: exposed above sea level (solid) and total (dashed) (based on Figure 4d in ref. [20]).

Geological records for exposed continents are rare for >3 Gyr ago. Spatially expansive exposure horizons can be identified in the Precambrian sedimentary record only after ~3 Gyr ago [20], and this is consistent with the abundant occurrence of submarine flood basalt magmatism in the Archean [21] and with the oxygen isotope record of shales [22]. Recent modeling of continental freeboard indicates that plate tectonics results in the net water flux from the oceans to the mantle at the rate of 3–4.5 × 10^14^ g/yr [20], and such positive net water influx has also been suggested by the global water cycle [23]. The Archean oceans could have been twice as voluminous as the current oceans, and even with the Armstrong model of continental growth, which assumes the present-day continental mass since 3.5 Gyr ago, the area of emerged continents would have been vanishingly small before ~3 Gyr ago [20], (Figure 1).

Knowledge of the surface environment in the early Archean and the Hadean remains elusive [24]. Though the oceans probably existed by 4.4 Gyr ago [25,26], the volume of continents and their exposed area are poorly constrained. As the operation of plate tectonics is not guaranteed in the early Earth, it becomes important to consider how surface environment was affected by a possible transition in the style of mantle convection. When such a transition took place is widely debated [27]; many geologists are content with the operation of plate tectonics at least back to ~3 Gyr ago [28], and some suggest that the geochemistry of zircon pushes its onset to >4.2 Gyr ago [29]. A recent geochemical modeling of Sm-Nd isotope systems suggests that plate tectonics could have started soon after the solidification of the putative magma ocean [30,31]. In any case, the tectonic regime before plate tectonics would probably have been stagnant lid convection, as it is the most natural style of thermal convection with strongly temperature-dependent viscosity [32]. In stagnant lid convection, hotspot volcanism, originating in the melting of mantle plumes, is the primary form of magmatism. The activity level of hotspot magmatism is ultimately controlled by core heat flow, part of which is manifested as mantle plumes. In the literature on early Earth, it is commonly assumed that high radiogenic heat production in the past resulted in higher heat flux [33,34]. However, the relation between heat production and heat flux is not so simple because, with the effect of mantle melting taken into account, a hotter mantle is likely to convect more slowly, rather than more rapidly [35]. The relationship between heat production and heat flux becomes even more counter-intuitive when core heat flux is involved [36].

**Figure 2:**
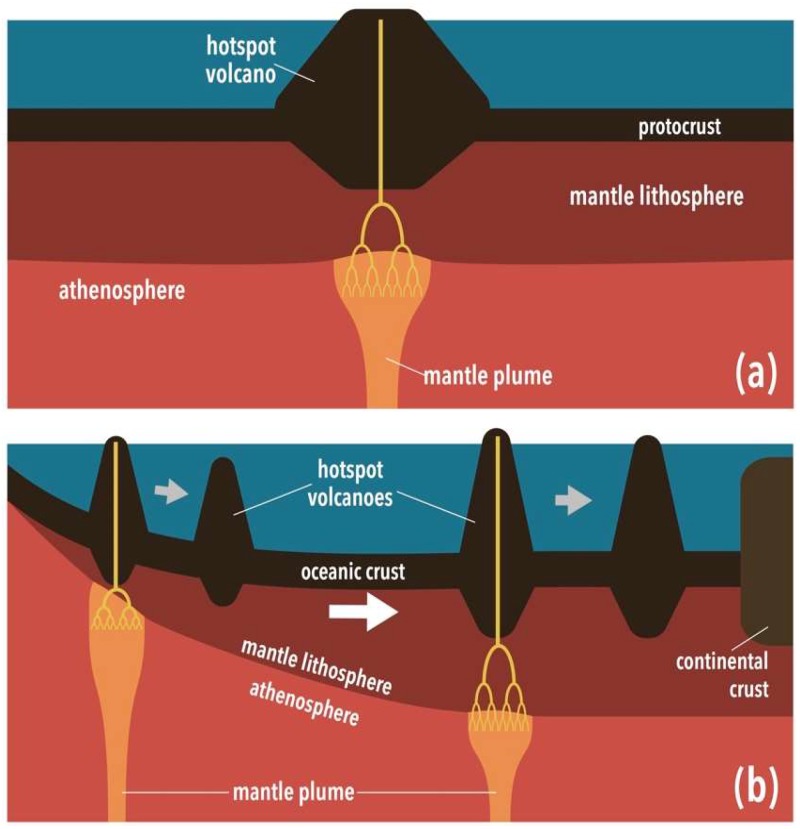
(**a**) Hotspot island formation in the stagnant lid regime of mantle convection. An island considerably larger than a typical hotspot island today can be constructed by continuous magma input from a stationary mantle plume. (**b**) Two contrasting fates of hotspot islands in early plate tectonics. Those formed on young ocean floor would have subsided as quickly as present-day hotspot islands, whereas those on older ocean floor could have been subaerial on a longer time scale because of subdued seafloor subsidence. See text for further explanation.

The thermal history of the core is not well constrained. Existing models of core evolution implicitly assume the continuous operation of plate tectonics since the beginning of Earth’s history [37], but the efficiency of core cooling would be lower without plate tectonics. At the same time, early core heat flux could have been higher than present even with stagnant lid convection, if the core was initially superheated. It would not be unreasonable to expect that the number of hotspot islands in the early Earth was similar to the present-day level (i.e., ~50 [38]). Even with twice as voluminous oceans, these hotspot islands could long have been subaerial, because, without plate motion, islands could keep growing with magma flux from stationary mantle plumes until they become subaerial, after which subaerial erosion starts to counteract growth (Figure 2a). Thus, prior to the onset of plate tectonics, exposed landmasses were probably limited to those numerous oceanic islands.

When plate tectonics started, continental crust began to exist in abundance, although most of it was probably under water until ~3 Gyr ago [20]. Some fraction of newly formed continental crust could have existed above sea level, at least temporally, due to crustal thickening resulting from continent–continent collision. Because of plate motion, which not only influences magma supply but also activates seafloor subsidence, the life-time of subaerial volcanoes reduced to the present-day level, that is, ~10 Myr, although a longer life-time (~100 Myr) could also have been possible for volcanoes formed on sufficiently old seafloor (Figure 2b). This is because of the fortuitous combination of the following two effects. First, past plate motion was likely to be slower than present [20,39], and the maximum seafloor age could have been as old as 400 Myr [40]. Second, with this longer time scale for oceanic plates, it becomes possible for them to be thermally equilibrated with internal heat production [41], and further seafloor subsidence would be prevented. Slow plate motion, coupled with the production of copious amounts of lava [42], would have helped to build more massive hotspot volcanoes on the early Earth, thereby increasing their chance to become subaerial. In the periods after 3.5 Gyr ago, there may have also been some limited areas of exposed continental crust above sea level.

The atmospheric composition was likely to have been drastically different before and after the transition to plate tectonics [43]. Before the transition, the atmosphere was likely similar today’s Venus (100 bar CO_2_ and hot, 100 °C). Carbon sequestration would have begun with the onset of plate tectonics. Long-lived islands during the stagnant lid phase were subject to Venus-like atmospheric conditions, while later they experienced a full spectrum of atmospheric conditions (hot, massive, CO_2_-rich; cool, periodic reducing-neutral atmospheric conditions; and finally a ~1 bar, N_2_-rich one).

With only volcanic islands protruding above sea level, these would have been the only land areas where WLPs could have existed on early Earth. The Hawaiian Islands have a total exposed surface area of 16,637 km^2^. Then, the assumption that the total number of hotspot volcanic islands on Earth >3.5 Gyr ago was similar that present currently (~50) implies a total exposed surface area for volcanic islands on the order of ~8x10^5^ km^2^. The Azores volcanic island chain today has ~0.4% of its total surface area (2.4 × 10^3^ km) covered by lakes, or 9.5 km^2^ [44]. Using this same relationship world-wide, suggests that on modern volcanic islands, lakes should occupy a total area of 480 km^2^.

## 3. WLPs Chemistry and Prebiotic Chemistry

Recent studies of mantle chemistry indicate that the Earth has a highly reducing deep mantle containing methane and hydrogen [45]. Today, a primordial helium isotopic component is present in gases from the Hawai‘i volcano Kilauae [46] and it has been recently suggested that abiotic CH_4_  +  H_2_ are constituents of the sublithospheric mantle [47]. It should be noted that modern day hot-spot volcanic gases are mainly oxidizing because of plume interactions with atmospheric oxygen, which would not have been the case on the young Earth. 

**Figure 3:**
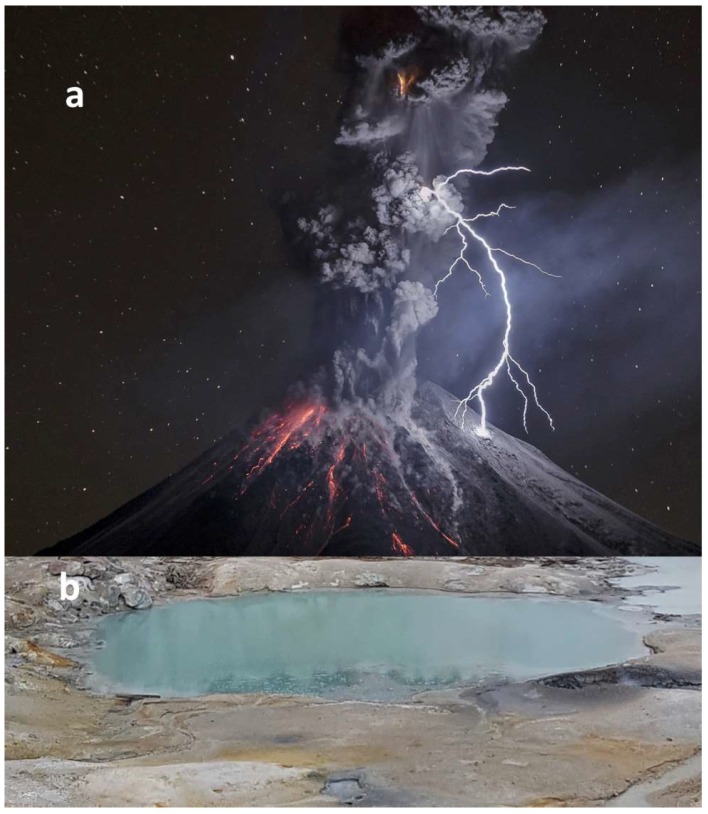
(**a**) Volcanic lightning associated with an eruption of the Colima Volcano in 2015 (photo credit: Sergio Tapiro); (**b**) a nearby “warm little pond” (WLP) on a volcano flank (Courtesy Ben K. D. Pearce).

Thus, on the early Earth hotspot volcanic emissions linked to hotspot deep-seated mantle plumes could have been rich in reduced gases. Localized prebiotic synthesis occurring in the lightning-rich gas emissions associated with these types of volcanoes could have been a robust source of organic compounds on the early Earth (Figure 3a). 

Evidence for this volcanic-island-based prebiotic synthesis of organic compounds has been provided from analyses of samples Stanley Miller archived from one of his classic 1950s spark discharge experiments [48,49]. This experiment utilized an apparatus configuration wherein a jet of water steam was directly injected into an electric discharge (Figure S1). A large variety of amino acids were found to be present in the archived residues Miller had saved from his original experiment. Included were several amino acids not produced in any of his other spark discharge experiments.

Additional results using a laboratory based volcanic lightning apparatus (Figure S2) have added further support for the potential of volcanic-island-based prebiotic syntheses [50]. Using a combination of reduced gases and washed volcanic ash from a Japanese volcanic eruption, it was found that traces of glycine were produced. It was hypothesized that in the discharges observed in the experiment, one of the components synthesized was HCN. It has been known for over a half-century that HCN can react to form HCN polymers that upon hydrolysis (carried out with the ash after the experiment), glycine and lesser amounts of other amino acids are produced.

We thus suggest that on volcanic islands on the early Earth, in association with lightning-rich eruptions emitting ash and reduced gases, the reagents needed for the synthesis of amino acids and other organic compounds could have been produced. The fallout from these eruptions then collected in WLPs or lakes on the flanks of the volcano where subsequent prebiotic synthesis reactions took place (Figure 3b).

## 4. Lake Waiau: A Modern Volcanic Island WLP?

**Figure 4:**
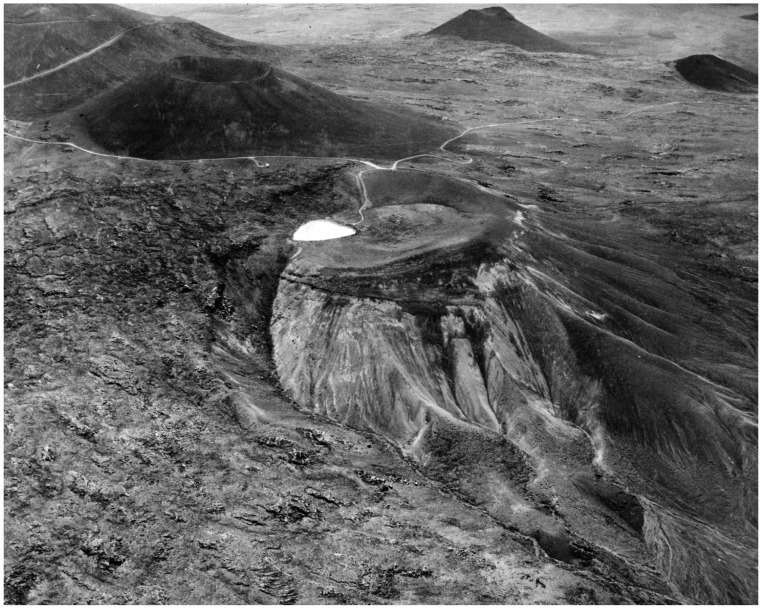
Aerial view taken in 1965 of ice-covered Lake Waiau in the Pu‘u Waiau cinder cone near the summit of the Mauna Kea volcano on the island of Hawai‘i (USGS Library, Denver; Photo KPA-N183].

A relevant modern analogue of a modern WLP is Lake Waiai (Figure 4) located in the Pu‘u Waiau cinder cone near the summit of presently dormant Mauna Kea volcano on the island of Hawai‘i (see [51,52,53,54,55] and references therein for various properties). This small (6000–7000 m^2^), shallow (3 m at full capacity) lake has existed for thousands of years. An ash layer in a sediment core from the lake indicates it was close to a local eruption that took place about 4500 BP. Lake water is supplied by local precipitation (mainly snow because of the 3870 m altitude) in the winter months. There is no evidence of any input of hydrothermal water into the lake. Melting snow water often overloads the lake’s holding capacity, which results in outflow into a nearby gully. In the summer, lake water evaporates and the lake size shrinks dramatically. In the winter the dissolved components of the lake are consistent with the sea spray enriched Hawaiian Island rainwater (Table S1). This is not surprising considering the Hawaiian Islands are ocean-encircled. However, the composition of sea spray (and thus rainwater) on the early Earth was likely much different than today because of the ancient ocean composition [14], being depleted in Mg^2+^ and enriched in Fe^2+^ relative to today. 

With Lake Waiau, summer evaporation is often extensive that in some years the lake has been nearly completely desiccated. Lake surface temperatures vary seasonally from ~0 °C to 13 °C and the lake generally freezes over during winter months. The lake had a pH of 8.6–9.1 from May–September, 1977. The bottom temperature of the lake is essentially a constant 6 °C throughout the year.

On the early Earth, Lake Waiau-like WLPs on volcanic islands could have been major sites for prebiotic synthesis reactions. Seasonal wet–dry cycles like on Lake Waiau today could have provided eutectic brines, concentrating reagents that would have then readily reacted to produce simple compounds, which in turn could have been converted into ones with increasingly complexity [55]. These types of processes may have provided the reactions and ingredients needed to eventually produce complex molecules with the properties of information storage and replication prone to random mutations, the hallmark of both the origin of life and evolution.

## 5. Conclusions

The scenario presented here has interesting consequences. With many hotspot volcanic islands on the early Earth and their associated WLPs and lakes, there could have been several different localities scattered across early Earth that allowed for various stages of prebiotic chemistry and chemical evolution. If chemical evolution proceeded to the point of producing complex molecules with the capacity for imperfect self-replication, there could have been a range of differing primitive biochemistries that emerged. As Darwin so eloquently noted “*Daily it is forced home on the mind of the geologist, that nothing, not even the wind that blows, is so unstable as the level of the crust of this* earth.” [56]. Volcanic islands would have eventually eroded and their diverse inventory and possible primitive biochemistries been released into the oceans, where competition for increasingly more effective biochemistries could have taken place. This thus would have begun the process of Darwinian evolution that eventually gave rise to the biochemistry we know today.

## Supplementary Materials

The following are available online at http://www.mdpi.com/2075-1729/8/4/55/s1, Figure S1: Miller’s “volcanic” spark discharge apparatus, Figure S2: Laboratory based volcanic lighting (courtesy Betty Scheu and Donald Dingwell), Table S1: Lake Waiau (LW) and Rain Water Composition Relative to Na+ = 1.0*.
