# Supplementary material for: Exposed Areas Above Sea Level on Earth >3.5 Gyr Ago: Implications for Prebiotic and Primitive Biotic Chemistry
Source: Life (Basel). 2018 Nov 4;8(4):55. doi: 10.3390/life8040055 (PMC6316429; doi:10.3390/life8040055)
Supplement: Supplementary file 1 [file life-08-00055-s001.pdf]

Supplementary Information (SI)

Table S1

Table S1: Lake Waiau (LW) and Rain Water Composition Relative to Na^{+} = 1.0*

	Na^{+}	K^{+}	Mg^{+2}	Ca^{+2}	Cl^{-}	SO_4^{-2}
**LW****	**1**	**0.59**	**0.64**	**0.59**	**2.89**	**0.19**
**Rain*****	**1**	**0.71**	**0.79**	**0.95**	**1.47**	**0.42**

***Lake water Na^{+} = 6.4 mg/l; rain water Na^{+} = 0.19 mg/l. Based on references S1 and S2. The lake also contains measurable levels of Cu, Fe, Ni and Zn (44).**
****Collected July 1972**
*****Collected at Saddle Road, Hawai'i, 29.3 km from sea, ~1700 m, Oct 19-20, 1956-57**

Figure S1: Miller's "volcanic" spark discharge apparatus

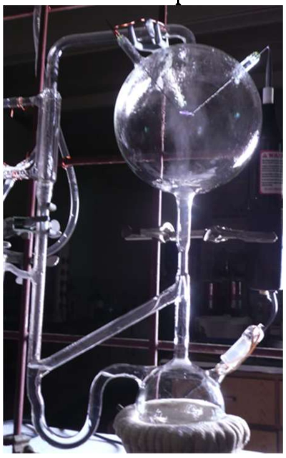

Figure S2: Laboratory based volcanic lighting (courtesy Betty Scheu and Donald Dingwell).

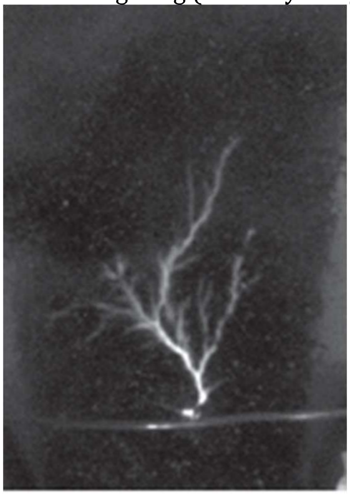

References

S1. J. A. Maciolek, J. A. (1982). "Lakes and lake-like waters of the Hawaiian Archipelago." *Occasional Papers of Bernice P. Bishop Museum*, XXV: 1-14.

S2. Eriksson, E. (1957). "The chemical composition of Hawaiian rainfall." *Tellus*: 509-520.
